# Effectiveness of Global Treatment Budgets for Patients With Mental Disorders—Claims Data Based Meta-Analysis of 13 Controlled Studies From Germany

**DOI:** 10.3389/fpsyt.2020.00131

**Published:** 2020-03-24

**Authors:** Fabian Baum, Olaf Schoffer, Anne Neumann, Martin Seifert, Roman Kliemt, Stefanie March, Enno Swart, Dennis Häckl, Andrea Pfennig, Jochen Schmitt

**Affiliations:** ^1^Center of Evidence-Based Health Care, Medizinische Fakultät Carl Gustav Carus, Technische Universität Dresden, Dresden, Germany; ^2^WIG2 Scientific Institute for Health Economics and Health System Research, Leipzig, Germany; ^3^Institute of Social Medicine and Health Services Research, Otto-von-Guericke-University Magdeburg, Magdeburg, Germany; ^4^Department of Psychiatry and Psychotherapy, Carl Gustav Carus University Hospital, Technische Universität Dresden, Dresden, Germany

**Keywords:** claims data, psychiatric health care, effectiveness, statutory health insurance, inpatient and outpatient treatment, setting approach, health care system, health services research

## Abstract

**Background:** Individuals with mental disorders need continuous and efficient collaboration between different sectors of care. In 2012, a new law in Germany enabled the implementation of novel budgets in psychiatry (flexible and integrated treatment = FIT). Hospitals implementing FIT programs have been evaluated in controlled cohort studies. We present first results based on a meta-analysis from 13 FIT hospitals.

**Methods/Design:** We undertook a series of claims-data-based controlled cohort studies. Data from over 70 statutory health insurance (SHI) funds in Germany were analyzed. All patients insured by any of the participating SHI funds and treated in one of the FIT hospitals for any of 16 predefined mental disorders were compared with matched control patients from routine care. The patient collective was subdivided into hospital-new and hospital-known patients. Analyses included utilization of inpatient care, day care, outpatient PIA (psychiatrische Institutsambulanz) care, outpatient care with established practitioners, and durations of sick leave. Individual treatment effects of the 13 FIT hospitals were pooled in a random-effects meta-analysis. Meta-regression analysis was used to explore potential reasons for heterogeneity in model effectiveness.

**Results:** The meta-analysis revealed a significant reduction by over 5 days of inpatient care in hospital-new patients in FIT hospitals compared to control hospitals. This effect was stronger among FIT hospitals with a preexisting FIT-like environment. There was no overall significant effect regarding sick leave between the two groups. Further meta-regression for hospital-new patients revealed a significantly reduced duration of sick leave by almost 13 days for patients in FIT hospitals with a preexisting FIT-like contract compared to FIT hospitals without such a contract.

**Conclusions:** This study suggests positive effects of FIT programs for patients with mental disorders pointing toward a shorter duration of inpatient treatment. Furthermore, contracts already existent prior to initialization of FIT programs appear to have facilitated the transition into the new treatment environment. For FIT hospitals without such contracts, supposedly there is a certain implementation phase for effects to be apparent. The results should still be interpreted with caution as this manuscript only covers the first year of the 5 year evaluation period in 13 of 18 FIT hospitals.

**Clinical Trial Registration:** This study was registered in the database “Health Services Research Germany” (trial number: VVfD_EVA64_15_003713).

## Background

Mental disorders are predominantly characterized by an early onset in patients' lives and by persisting over long periods of time (1, 2). However, utilization of treatment in psychiatric patients is low with a long delay between the onset of illness and initial treatment (3–5). Efficient patient-centered treatment of these disorders demands for a continuous and close collaboration between different sectors and professions of care (3, 6). As early as in the 1970s, the Study Commission of the German Bundestag urged for new approaches to health care particularly aiming at—whenever possible—a preference of outpatient over inpatient treatment and, in addition, a regionalized health care (7).

However, current standard care for patients with mental disorders in Germany is still characterized by a strong focus on inpatient treatment (8). Furthermore, the German healthcare system currently suffers from insufficient interfaces between different sectors of health care, particularly in the field of psychiatric care (9). This situation results in an inadequate integration of inpatient and outpatient treatment, psychotherapy, and psychosocial services and might even obstruct joint care approaches involving multiple medical specialists (10, 11). Additionally, there is also a great fragmentation of the financing system in German psychiatric health care: budgets for inpatient and day care services are strictly separated from the budget of the psychiatric outpatient departments (PIA, psychiatrische Institutsambulanz). These departments treat patients in need of particularly intensive and complex near-hospital care due to the nature, severity, or longevity of the mental disorder. This financial separation constitutes an additional obstacle for efforts toward an efficient trans-sectoral treatment (12), potentially even resulting in misguided incentives, such as maximizing inpatient occupancies by admitting as many patients as possible with the highest possible retention time (13).

In response, there have been a number of initiatives promoting new financing budgets (from now on called global treatment budgets) aiming to promote patient-centered, cross-sectoral health care for mentally ill patients (14–17). The most recent taking on this issue is based on a German law launched in 2012 [§ 64b, German Social Code V, (18)]. It enabled statutory health insurance (SHI) funds, together with selected hospitals, to jointly establish contracts that represent a hybrid installment of both capitation budget (19–22) and block contract (23–25). According to these contracts, each hospital has an overall fixed annual budget for all patients including inpatient care, day care, and outpatient care. This budget covers all treatment expenses leaving room for the provider to apply an individual treatment strategy. These novel programs will from now on be called FIT programs [FIT = flexible and integrated treatment (26, 27)], and hospitals implementing this type of programs will from now on be referred to as FIT hospitals. FIT hospitals are free to tailor specific models of care that suit the regional peculiarities and meet the needs of community members (28). Thus, they tend to differ tremendously in terms of starting conditions as well as treatment and process structures. All of the FIT programs, however, share the common goal of providing a continuous, flexible, and integrated treatment to patients.

As part of the legal requirement, the authors of this manuscript are in charge of conducting an evaluation of almost all FIT hospitals. This study was, in accordance with requests formulated in prior research (6, 29), designed to provide a standardized evaluation procedure. In this overall evaluation study, called EVA64, patients being treated in 1 of a total of 18 FIT hospitals constitute the intervention group (IG) and are compared to patients from routine care hospitals, which form the control group (CG).

This manuscript describes first results of effectivity measures based on meta-analyses and meta-regressions over the intermediate reports of the 13 FIT hospitals that have been evaluated so far. Analyses included utilization of inpatient care, day care, outpatient PIA care, as well as outpatient care with established practitioners. Furthermore, we analyzed durations of sick leave. Based on the primary goals of FIT programs, we hypothesized a reduction in inpatient care and in the duration of sick leave among patients in FIT hospitals compared to patients from control hospitals.

## Methods

### Study Design

We undertook a series of controlled cohort studies (1 for each of the 13 FIT hospitals evaluated so far) employing a pre–post design based on health insurance data from each of the intervention hospitals and its matched control hospitals (30). We used anonymized patient data from German statutory SHI funds covering a total time span of 2 years with 1 year prior and 1 year subsequent to inclusion in the evaluation. FIT programs started between January 2013 and December 2014 in all analyzed hospitals. FIT hospitals differed with respect to starting conditions: four hospitals transitioned from standard care to FIT, while the remaining nine hospitals transitioned into the FIT environment with a preexisting contract. Those contracts had goals similar to those of the FIT programs (=FIT-like structures) and could include a regional budget for mental health care, a contract for integrated care, or both options combined (17).

### Study Population

All patients insured by any of the participating SHI funds and treated in one of the FIT hospitals (IG) for any of 16 predefined mental disorders (see Table 1) were compared with control patients from routine care (CG). For each individual hospital population, subcohorts of hospital-known and hospital-new patients were defined. Hospital-new patients had no contact to the psychiatric ward or PIA in the corresponding FIT or control hospital in the 2 years prior to being included. Hospital-known patients had to have at least one such contact during those 2 years. Our reasoning behind the subdivision of the cohort into hospital-new and hospital-known patients was that potential intervention effects would have a different impact for IG/CG patients who already had a treatment history at an FIT/control hospital compared to IG/CG patients whose initial treatment took place after the onset of the FIT intervention. This differentiation gains even more importance considering that 9 out of 13 FIT hospitals already have had specific contracts that to a certain extent exhibited FIT-like structures prior to initiation of the FIT programs. These already preexisting contracts are likely to have facilitated the transition into the new FIT environment and could have already forestalled some of the intended intervention effects before FIT initiation (15, 17, 31, 32). Hence, we expected more unbiased intervention effects to occur in the subcohort of hospital-new patients.

**Table 1:** Inclusion criteria, diagnoses, International Classification of Diseases, 10th revision (ICD−10).

**ICD-10**	**Diagnosis**
F00	Dementia
F01	Vascular dementia
F02	Dementia in other diseases classified elsewhere
F03	Unspecified dementia
F07	Personality and behavioral disorders due to brain disease, damage, and dysfunction
F10	Mental and behavioral disorders due to use of alcohol
F20–F29	Schizophrenia, schizotypal, and delusional disorders
F30–F39	Mood (affective) disorders
F43	Reaction to severe stress, and adjustment disorders
F45	Somatoform disorders
F40–F48	Neurotic, stress-related, and somatoform disorders
F50	Eating disorders
F60.31	Specific personality disorders of type borderline
F70–F79	Mental retardation
F84	Pervasive developmental disorders
F90–F98	Behavioral and emotional disorders with onset occurring in childhood and adolescence

### Matching on Hospital and Patient Level

To minimize the likelihood of selection bias on the provider and patient level, a two-level matching algorithm was applied. On the hospital level, we were primarily concerned with assigning comparable control hospitals to each FIT hospital. Therefore, up to 10 control hospitals were allocated in ranking order to each FIT hospital based on a priori defined knockout criteria (i.e., same region, institutionalized structures, such as specialist departments, and PIA), criteria based on patients (i.e., number of cases per diagnosis) with a weighting of 50%, structural features of hospitals (e.g., number of beds or number of personnel) with a weighting of 25%, and regional factors (e.g., unemployment rate, household income) with a weighting of 25%. More details can be found in the already-published study protocol (33). Once we identified best-fitting control hospitals for each FIT hospital, we applied a second matching algorithm on the level of patients. This was done in order to reduce the impact of possible confounding variables by leveling out IG and CG patient distributions regarding these exact variables. Patients were matched exactly according to the variables: year of study inclusion, hospital-known and hospital-new patient, and type of mental disorder diagnosed at study inclusion. Thus, for these variables, twin pairs of IG and CG patients had to exhibit the exact same value. Furthermore, propensity score matching was applied on variables sex, age at study inclusion, and health care utilization before study inclusion (amount of inpatient care, day care, and outpatient utilization in PIA and established practitioners, all in the area of mental health care). The propensity matching procedure is based on a patient's probability (i.e., propensity score) of group membership (IG/CG), which is calculated by logistic regression for the entire population. Patients' propensity scores were utilized to determine twin pairs of IG and CG members based on a nearest neighbor algorithm (caliper = 0.25 standard deviation, without replacement). Hence, each patient of the IG was assigned its best-fitting twin from the CG based on the least difference in value defined by the propensity score. Note that one CG can contain patients from more than one hospital.

### Data and Outcomes

The standardized dataset provided by the participating SHI funds covered inpatient care, day care, and outpatient care (diagnoses and procedures) including PIA and established practitioners, pharmaceutical and non-pharmaceutical treatments (i.e., psychotherapy), and information on sick leave. As all analyzed data were anonymous, the ethical committee of the University of Magdeburg confirmed that no ethical approval was necessary. Data were handled, analyzed, and reported according to Good Epidemiological Practice (GEP) (34), Good Practice of Secondary Data Analysis (GPS) (35), and the German Reporting Standard for Secondary Data Analyses, Version 2 (STROSA 2) (36).

Outcome parameters between IG and CG were compared with respect to the patient–individual pre-time, which was 1 year prior to study entrance. We report first results from inception until the end of the first year of intervention in 13 out of the total 18 FIT hospitals. Primary outcomes were *duration of inpatient care* and *sick leave*. The first outcome describes the average cumulated length of hospitalization days. For the parameter *sick leave*, we aggregated the average cumulated number of days in sick leave. The inclusion diagnosis, in this case, was based on inpatient and outpatient sick leave prescriptions. In addition, every day a patient spent in inpatient care was counted as sick leave day. The analysis was restricted to patients with “member” status as reported by the corresponding SHI. This was done in order to exclude retirees and people who were not capable of working, who have a different status of health insurance. Secondary outcomes included the cumulated duration of day care (number of days) as well as the cumulated number of contacts in outpatient care. We split outpatient care into (i) contacts to the PIA and (ii) contacts to established medical specialists for psychiatry or psychotherapy. We assumed that a potentially reduced inpatient care in the initial treatment phase could be compensated for through either day care or outpatient treatment. To this end, we analyzed day care and outpatient utilization (for each PIA and established practitioner) in addition to the duration of inpatient care. To further analyze the association of duration of inpatient and duration of day care treatment for the cohort of hospital-new patients, we calculated a Pearson correlation coefficient on the level of hospitals between these two measures.

### Statistical Analysis on Single Hospital Level

Estimators for outcomes of interest were computed using generalized linear Poisson models. Models contained variables for factors *group* (IG vs. CG) and *time* (pre vs. 1st year after inclusion into the study) as well as an additional variable for the interaction term *group x time*. As the interaction term approximates the difference-in-difference (DiD) coefficient, and hence the actual effect of treatment, regression coefficients for that interaction term were later used in the meta-analysis. The DiD coefficient compares the average change over time in the outcome variable for the IG in contrast to the average change over time for the CG. Thus, greater changes over time in the IG compared to the CG are associated with a positive DiD coefficient and vice versa.

### Meta-Analysis Over 13 Evaluated FIT Hospitals vs. Controls

The DiD coefficients for every single FIT hospital were used in a meta-analysis regarding the primary outcomes *duration of inpatient care* as well as duration of *sick leave*, as well as the secondary outcomes *duration of day care* and *amount of outpatient contacts* for each PIA and established practitioner, respectively. The meta-analysis was done utilizing the R package metaphor (37). The heterogeneity measure I^2^ between the individual entities was expected to be high since hospitals recruited different populations and had varying starting conditions. Taking these considerations into account, the pooled estimator was modeled as random effect. The heterogeneity parameter τ^2^ was estimated using the *DerSimonian–Laird* estimator. Due to insufficient pre-period data of outpatient contacts in the PIA in 6 of the 13 FIT hospitals, this meta-analysis only compared mean differences in the first year after onset of the project. Regarding contacts to medical specialists, we were able to apply the usual procedure of calculating a pooled effect over individual DiD estimates. In addition, we carried out a meta-regression for selected outcomes to investigate the impact of preexisting FIT–like structures. Therefore, we included a predictor variable into the meta-regression that dummy-coded hospitals with a preexisting contract (0 = no existing contract vs. 1 = existing contract). The result of the meta-regression would give an estimate on how FIT hospitals with preexisting contracts would contrast against those without such a contract. All statistical analyses were done using statistical software R V. 3.3.2 (38).

## Results

### Baseline Characteristics

The number of patients per group ranged between 153 and 852 in hospital-new, and 164 and 1,207 in hospital-known patients in different hospitals. The overall cohort consisted of 26,398 patients (13,199 each in IG and CG) with 12,468 (6,234 each) being hospital-new and 13,930 (6,965 each) being hospital-known. Mean age and sex were highly comparable between IG and CG (see Table 2). Throughout all hospitals, regardless of group, more women were included (56%).

**Table 2:** Characteristics of patients of all hospitals at the time of study inclusion.

	***N***		**Mean age (years)**		**Percentage women**	
	**IG**	**CG**	**IG**	**CG**	**IG%**	**CG%**
**Cohort of hospital-new patients**	**6,234**	**6,234**	**50.6**	**50.6**	**56**	**56**
A	770	770	41.9	41.9	62	62
B	153	153	49.9	50	69	61
C	748	748	47.7	48.3	58	57
D	637	637	50	49.4	58	60
E	426	426	52.3	53.8	55	54
F	594	594	50.9	50.6	55	53
G	375	375	52.3	51.9	52	54
H	507	507	50.6	49.8	58	59
I	221	221	54.4	52.8	44	45
J	852	852	53.3	52.3	56	57
K	204	204	50	50.8	55	57
L	289	289	52.8	52	51	54
M	458	458	51.4	53.6	58	52
**Cohort of hospital-known patients**	**6,965**	**6,965**	**51.5**	**51.4**	**56**	**56**
A	663	663	45.7	47.4	56	58
B	245	245	53.6	51.2	68	67
C	1,207	1,207	49.2	50.3	55	58
D	705	705	48.4	49	63	59
E	496	496	52.2	52.6	52	55
F	469	469	49.6	51.6	51	54
G	318	318	52.6	53	42	46
H	486	486	50.8	49.6	55	53
I	259	259	53.6	53.6	49	48
J	981	981	56.7	56.8	58	59
K	337	337	55.5	53.7	63	60
L	164	164	49.3	46	61	51
M	635	635	51.8	53.3	54	55

*IG, intervention group (patients from FIT hospital A-M); CG, control group (patients from corresponding control hospitals)*.

### Descriptive Data

#### Duration of Inpatient Care

Among hospital-new patients, the number of days spent in hospital was low before inclusion and sharply increased within the first year after inclusion (see Table 3). However, across all FIT hospitals, the sharp increase in hospital stay was lower in the IG (from 2 to 15.6 days) compared to the CG (from 2.2 to 21.3 days).

**Table 3:** Duration of inpatient care.

	**IG**		**CG**	
	**pre**	**1^**st**^ yr**	**pre**	**1^**st**^ yr**
Cohort of hospital-new patients (*n*)	6,234		6,234	
Cumulated duration (days) of inpatient care per patient	mean (sd)			
A	3.9 (17.3)	13.3 (27.0)	3.8 (16.9)	20.0 (31.1)
B	1.4 (6.6)	12.4 (24.0)	3.6 (19.6)	24.9 (39.8)
C	2.2 (14.4)	9.4 (22.1)	1.8 (9.7)	13.9 (30.3)
D	2.7 (15.5)	16.6 (25.9)	1.6 (10.3)	23.7 (34.3)
E	2.0 (12.7)	6.4 (15.0)	3.2 (21.0)	17.3 (27.0)
F	1.4 (10.5)	18.1 (24.0)	1.9 (12.5)	23.4 (36.4)
G	2.2 (14.8)	17.2 (32.9)	2.3 (13.0)	21.0 (32.3)
H	1.1 (8.3)	11.3 (28.4)	1.5 (9.4)	23.9 (36.5)
I	1.0 (4.6)	20.0 (23.2)	0.4 (2.7)	20.6 (25.6)
J	1.7 (12.9)	10.7 (25.8)	2.0 (10.5)	19.6 (30.6)
K	1.8 (11.3)	18.6 (34.2)	1.2 (8.3)	17.6 (29.5)
L	2.6 (14.2)	30.0 (43.8)	2.9 (14.5)	25.9 (34.6)
M	1.6 (13.3)	19.4 (26.3)	2.7 (16.3)	25.2 (34.9)
Grand mean over all hospitals	2.0	15.6	2.2	21.3
Grand mean of only FIT hospitals with preexisting FIT-like contract	2.0	13.9	2.2	20.9
Grand mean of only FIT hospitals without preexisting FIT-like contract	1.9	19.7	2.2	22.1
Cohort of hospital-known patients (*n*)	6,965		6,965	
Cumulated mean duration (days) of inpatient care per patient	mean (sd)			
A	8.3 (21.8)	9.8 (27.2)	9.2 (24.7)	17.9 (33.6)
B	11.0 (25.5)	10.1 (21.6)	13.5 (33.2)	13.7 (27.7)
C	8.9 (27.7)	7.2 (24.5)	15.8 (38.4)	12.4 (32.4)
D	7.5 (22.3)	10.4 (24.8)	13.4 (31.5)	15.2 (31.3)
E	5.7 (16.4)	7.4 (21.4)	11.6 (31.2)	14.3 (30.4)
F	14.9 (27.1)	18.0 (31.3)	20.4 (39.7)	26.1 (43.2)
G	11.1 (31.9)	14.1 (31.1)	13.1 (30.0)	19.3 (34.1)
H	8.8 (25.3)	8.8 (23.2)	14.3 (33.1)	17.0 (34.5)
I	8.2 (21.7)	10.5 (22.3)	9.3 (24.5)	13.4 (32.0)
J	12.4 (34.2)	10.2 (30.9)	11.4 (29.8)	12.5 (28.0)
K	11.2 (29.6)	11.8 (32.1)	9.9 (26.8)	12.2 (28.8)
L	20.4 (37.0)	32.9 (46.6)	21.8 (32.9)	24.7 (39.9)
M	13.2 (31.2)	14.1 (35.0)	16.6 (32.7)	15.0 (30.5)
Grand mean over all hospitals	10.9	12.7	13.9	16.4
Grand mean of only FIT hospitals with preexisting FIT-like contract	9.4	10.7	13.4	16.6
Grand mean of only FIT hospitals without preexisting FIT-like contract	13.6	16.3	14.7	16.2

*IG, intervention group (patients from FIT hospital A-M); CG, control group (patients from corresponding control hospitals); pre, 1 year before inclusion; 1^st^ yr, 1 year after inclusion*.

Hospital-known patients had fewer inpatient days in the IG compared to the CG. The increase in inpatient care days was slightly lower in the IG (10.9 to 12.7 days) compared to the CG (from 13.9 to 16.4 days; Table 3).

### Duration of Sick Leave

The amount of days of sick leave sharply increased within the first year after inclusion (Table 4). There was no notable overall difference in the increase of days of sick leave between the two groups (see Table 4).

**Table 4:** Duration of sick leave due to inclusion diagnosis.

	**IG**		**CG**	
	**pre**	**1^**st**^ yr**	**pre**	**1^**st**^ yr**
Number of hospital-new patients capable of working (*n*)	3,433		3,430	
Cumulated number of days in sick leave	mean (sd)			
A	33.8 (75.3)	70.8 (106.8)	29.5 (69.7)	75.3 (104.1)
B	27.5 (74.4)	83.6 (108.3)	16.1 (41.1)	75.3 (85.2)
C	15.9 (45.4)	57.8 (96.9)	13.2 (32.5)	59.9 (92.4)
D	25.9 (62.9)	58.2 (86.2)	18.5 (49.3)	67.9 (93.2)
E	19.6 (64.2)	58.6 (92.8)	13.6 (42.5)	57.4 (85.3)
F	20.3 (50.0)	67.0 (89.6)	16.1 (42.9)	63.5 (91.7)
G	19.1 (47.2)	78.8 (102.1)	20.0 (56.0)	63.8 (100.4)
H	16.6 (40.6)	58.5 (78.1)	13.1 (43.4)	61.1 (91.2)
I	15.6 (39.4)	66.2 (91.5)	10.3 (28.9)	67.2 (90.1)
J	17.7 (49.1)	61.5 (96.6)	16.7 (45.7)	57.8 (83.2)
K	19.7 (54.5)	75.2 (103.0)	18.4 (57.7)	57.4 (86.1)
L	15.3 (40.3)	70.0 (74.9)	31.8 (70.0)	62.6 (80.8)
M	22.8 (56.8)	72.5 (92.6)	25.5 (56.4)	82.6 (96.3)
Grand mean over all hospitals	20.8	67.6	18.7	65.5
Grand mean of only FIT hospitals with preexisting FIT-like contract	21.6	66.6	16.7	65.7
Grand mean of only FIT hospitals without preexisting FIT-like contract	18.9	69.8	23.1	65.1
Number of hospital-known patients capable of working (*n*)	3,183		3,116	
Cumulated number of days in sick leave	mean (sd)			
A	36.4 (75.2)	36.5 (74.5)	37.5 (77.0)	49.7 (86.3)
B	60.8 (82.3)	67.8 (102.7)	59.5 (93.0)	62.3 (90.2)
C	28.5 (63.5)	28.2 (63.7)	29.9 (62.8)	30.2 (61.9)
D	28.6 (63.7)	31.0 (65.8)	32.9 (64.7)	31.2 (64.1)
E	31.6 (67.9)	30.7 (57.4)	30.8 (59.2)	42.0 (79.2)
F	29.7 (51.9)	39.3 (72.5)	40.0 (66.5)	42.1 (59.4)
G	35.8 (74.5)	35.7 (71.0)	27.3 (50.7)	40.1 (72.4)
H	30.4 (55.8)	28.6 (61.9)	32.9 (67.1)	30.8 (57.8)
I	18.3 (46.1)	21.7 (35.5)	27.0 (54.2)	29.9 (61.2)
J	28.7 (58.6)	29.8 (66.7)	31.2 (60.2)	40.3 (75.5)
K	39.4 (73.9)	45.4 (90.7)	20.4 (50.8)	18.1 (44.0)
L	58.3 (75.5)	72.9 (83.8)	64.0 (85.1)	71.5 (92.3)
M	46.2 (78.2)	37.2 (70.5)	44.3 (73.7)	37.7 (69.2)
Grand mean over all hospitals	36.3	38.8	36.7	40.5
Grand mean of only FIT hospitals with preexisting FIT-like contract	33.3	35.5	35.3	39.8
Grand mean of only FIT hospitals without preexisting FIT-like contract	43.1	46.3	40.0	41.9

*IG, intervention group (patients from FIT hospital A-M); CG, control group (patients from corresponding control hospitals); pre, 1 year before study inclusion; 1^st^ yr, 1 year after study inclusion; capable of working, health insurance status, member (own insurance and not through family member, no pensioner)*.

The aggregated average number of days of sick leave was lower in the IG compared to the CG in the first year of the evaluation. Overall, there was a slight increase in the duration of sick leave in both groups with the increase in the IG being slightly lower compared to the CG.

### Meta-Analysis

#### Duration of Inpatient Care

**Figure 1:**
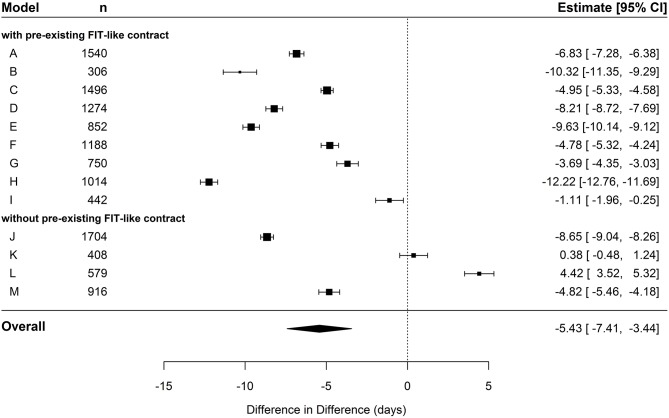
Forest plot depicting effects of cumulated duration of inpatient care in hospital-new patients.

In the cohort of hospital-new patients, the pooled effect of the overall difference in cumulative number of inpatient care days per person between IG and CG exceeded the significance level (*p* < 0.001). The pooled DiD showed an average decrease of 5.4 days (95% CI: −7.41; −3.44) in the IG compared to the CG (see Table 5 and Figure 1). The meta-regression unfolded that this effect was especially driven by FIT hospitals that already had a preexisting contract. When contrasted against the remaining four FIT hospitals without such a contract, hospitals with a preexisting contract had a significantly reduced DiD of ~4.7 days in the meta-regression (see Table 6).

**Table 5:** Pooled estimates of meta-analysis over individual DiD estimates for different outcomes.

**Outcome**	**Estimate**	**Lower CI**	**Upper CI**	***p***	**I^**2**^**
**Hospital-new patients**					
Number of days in inpatient care	**−5.43**	**−7.41**	**−3.44**	**0.000**	**99.39**
Number of days in day care	2.12	−0.76	5.00	0.15	99.89
Number of days in sick leave	−0.02	−5.51	5.47	0.994	99.36
Number of outpatient contacts (PIA)	0.43	−0.57	1.43	0.40	99.56
Number of outpatient contacts (established practitioner)	−0.06	−0.54	0.43	0.81	96.17
**Hospital-known patients**					
Number of days in inpatient care	−0.77	−2.5	0.97	0.386	99.08
Number of days in day care	0.13	−0.68	0.94	0.75	98.94
Number of days in sick leave	−1.27	−5.18	2.63	0.523	98.86
Number of outpatient contacts (PIA)	−0.12	−1.75	1.51	0.89	99.54
Number of outpatient contacts (established practitioner)	0.29	−0.07	0.46	0.15	92.86

*Bold values represent significant effects*.

**Table 6:** Meta-regression effect sizes of FIT-like pre-contracts (existing vs. nonexisting) in different outcomes.

**Outcome**	**Slope coefficient**	***p***
**Hospital-new patients**		
Number of days in inpatient care	**−4.67**	**0.041**
Number of days in day care	1.56	0.647
Number of days in sick leave	**−12.89**	**0.022**
**Hospital-known patients**		
Number of days in inpatient care	−3.58	0.079
Number of days in sick leave	−5.51	0.442

*Bold values represent significant effects*.

**Figure 2:**
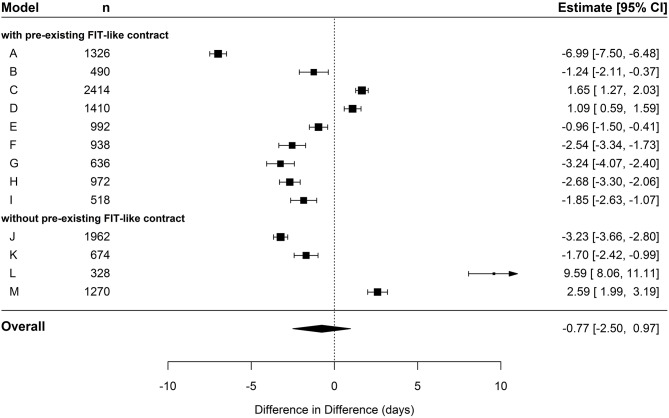
Forest plot depicting effects of cumulated duration of inpatient care in hospital-known patients.

In the cohort of hospital-known patients, the pooled estimate displayed a decrease of 0.77 days (95% CI: −2.5; 0.97) in the IG compared to the CG (see Table 5 and Figure 2). However, this effect did not exceed the significance level. The following meta-regression revealed no significant difference in FIT hospitals that already had a preexisting contract compared to hospitals without a contract (see Table 6). In general, coefficients of individual DiD effects in the cohort of hospital-known patients revealed smaller absolute values compared to the cohort of hospital-new patients, especially in FIT hospitals with preexisting FIT-like contracts. This is mainly due to already-existent baseline differences in the pre-period between IG and CG. Those baseline differences might lead to possible ceiling effects by leaving not much room for further improvement in the post-period. This pre-period difference between the two groups was not that pronounced in FIT hospitals without already-existing FIT-like structure (see Table 3).

#### Cumulated Number of Contacts in Outpatient Care

Analysis for outpatient contacts in PIA or with an established practitioner did not yield statistically significant results in both hospital-new and hospital-known patients. Neither the number of contacts in the PIA nor the number of contacts to medical specialists were considerably increased in intervention patients compared to patients of the CG (see Table 5).

#### Cumulated Duration in Day Care

**Figure 3:**
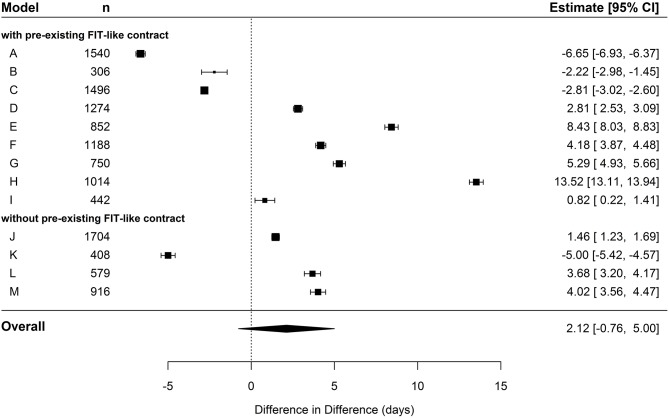
Forest plot depicting effects of cumulated duration of day care in hospital-new patients.

In the meta-analysis of cumulated day care duration for hospital-new patients, the pooled estimate did not exceed the significance level as well (*p* = 0.15, see Table 5). However, the IG showed a trend toward a higher utilization (~2 days) compared to the CG (see Figure 3).

**Figure 4:**
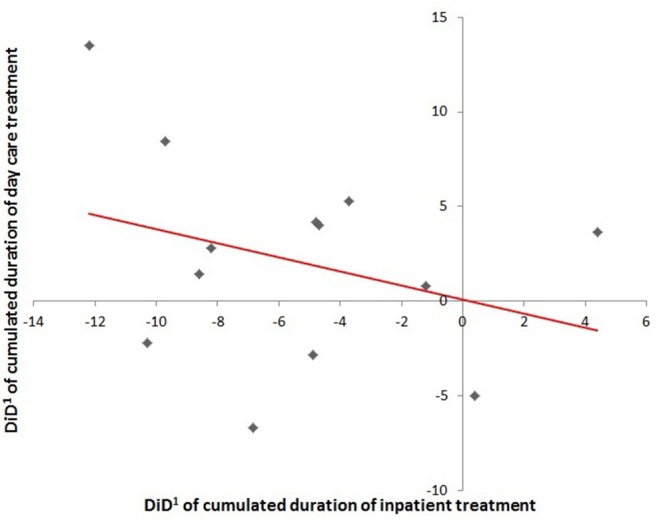
Regression line depicting the association between duration of inpatient treatment and duration of day care treatment in hospital-new patients (n.s.) ^1^ DiD, difference-in-difference.

We identified a negative correlation coefficient of −0.31 between the utilization of inpatient care and day care. The more inpatient treatment days were reduced in the IG of a specific FIT hospital, the more the number of days in day care treatment was increased compared to the CG and vice versa (see Figure 4). This correlation did not yet exceed the significance level (*p* = 0.15), which is mainly due to the lack of statistical power as only data pairs from 13 FIT hospitals were available up to that point. In hospital-known patients, there was no significant difference in day care utilization between IG and CG.

#### Duration of Sick Leave

**Figure 5:**
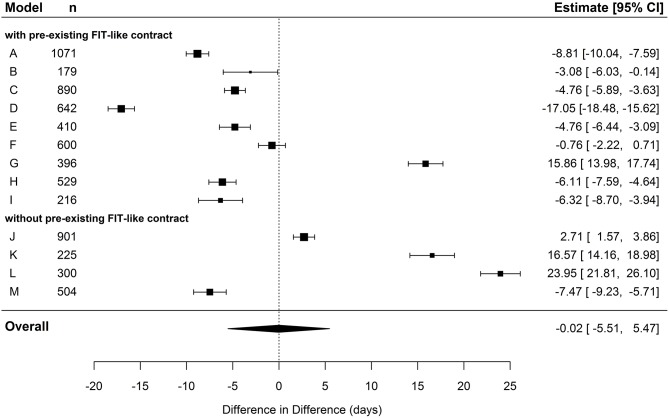
Forest plot depicting effects of cumulated duration of sick leave in hospital-new patients.

In the cohort of hospital-new patients, there was no significant pooled effect in the overall difference in cumulative number of sick leave days per person between IG and CG (pooled estimate = −0.02; *p* = 0.994, see Table 5 and Figure 5). There is a significant individual effect in almost every FIT hospital contrasted against its respective CG. However, the direction of these effects is quite diverse. When contrasting hospitals who already had a preexisting FIT-like contract against the remaining hospitals without such a contract as reference in the meta-regression, we found a massive effect of an increased negative DiD (see Table 6). Hence, whether or not an FIT hospital already had a preexisting FIT-like structure plays a huge role in explaining the large heterogeneity of the individual DiD effects. Hospitals with a preexisting contract had a reduced duration of sick leave of almost 13 days compared to hospitals without such a contract. Based on this result, we additionally performed a meta-analysis only including the nine FIT hospitals with a preexisting contract. The pooled estimate did not also exceed the significance level, but, however, showed a stronger tendency toward a reduced duration of sick leave of over 4 days in IG patients compared to CG patients (pooled estimate = −4.34; *p* = 0.076).

**Figure 6:**
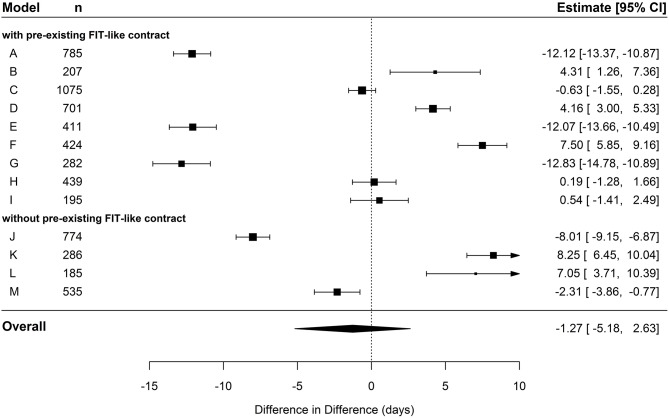
Forest plot depicting effects of cumulated duration of sick leave in hospital-known patients.

In the cohort of hospital-known patients, the pooled estimate displayed a decrease in days of sick leave of 1.27 days in the IG compared to the CG (see Table 5 and Figure 6). This effect did not exceed the significance level. The meta-regression revealed no significant difference in FIT hospitals that already had a preexisting contract compared to hospitals without a contract (see Table 6).

## Discussion

The EVA64-study evaluates 18 different nationwide FIT hospitals, which aim to improve the health care of patients with mental disorders in Germany on the basis of novel global treatment budgets. This manuscript presents results of the EVA64-study based on a meta-analysis over the first intermediate evaluative reports of 13 of these FIT projects.

The meta-analysis showed a significantly lower increase in the cumulated duration of inpatient care in hospital-new intervention patients (IG) compared to control patients (CG) indicating positive effects on one of the primary goals of the FIT intervention (30). This effect was even stronger when only considering FIT hospitals that transitioned from an already-existing FIT-like contract into the new environment. The overall sharp increase in inpatient care from pre-period to the end of the first year in both groups was expected, as the hospital-new patient's inclusion diagnosis is likely to be an incident diagnosis resulting in a high initial demand of care. In hospital-known patients, there was already a substantial baseline difference between IG and CG in those FIT hospitals. This was not the case when only considering FIT hospitals without any preexisting contract in the pre-period. These results may lead to various conclusions with high impact for the organization of clinical care in mental health settings. First, the FIT intervention is effective in terms of reducing fully inpatient care. Secondly, preexisting FIT-like contracts in the pre-period may have already forestalled some of the intended effects. This presumption is supported by findings from other authors who explicitly examined the effects of some of these contracts in various hospitals (16, 17, 39). On the other hand, pre-contracts possibly facilitated a faster and smoother implementation of the new FIT environment. Intervention hospitals starting from scratch are likely to undergo a longer transition and implementation period. Thus, potential FIT effects are not likely to be already present in the first year after onset in those clinics. Interestingly, we found tendencies of overall increased day care duration but not an increase in outpatient contacts in hospital-new patients. Moreover, there seems to be a correlation between the reduction of inpatient care and day care. The more inpatient care is reduced in an FIT hospital, day care treatment increases. This may give rise to the interpretation that reduced inpatient days in FIT hospitals are rerouted into day care treatment but not necessarily into outpatient treatment *per se*.

Analogously to inpatient treatment, there was a sharp increase in sick leave duration from pre-period to the end of the first year in both groups. As hospital-new patients' inclusion diagnosis is likely to be an incident diagnosis, this may lead to an increased number of sick leave days. On first sight, there seems to be no considerable difference regarding trends in sick leave duration between FIT patients and control patients. The pooled estimator showed no significant difference in the average cumulated number of sick leave days between IG and CG in both hospital-new and hospital-known patients. With the individual effects being very heterogeneous, a further meta-regression for hospital-new patients revealed that sick leave duration was significantly lower by almost 13 days in FIT hospitals with an already preexisting FIT-like contract in the pre-period compared to FIT hospitals without such a contract. A concluding meta-analysis containing only FIT hospitals with an FIT-like contract in the pre-period showed a clear tendency of reduced sick leave duration in intervention patients compared to control patients, albeit without being significant.

## Strengths and Limitations

The study presented gathers data from FIT hospitals from locations all over Germany in total representing more than 300,000 patients (40). Yet, follow-up of the results described is still limited to only the first year of the all in all five-year evaluation period. Thus, this manuscript can only cover potential short-term effects, leaving out effects that occur only over a longer time period. Further reports with data spanning over a longer observation period will reveal whether positive intervention effects will be present or strengthened in FIT hospitals, particularly in those without any preexisting contract. So far, only 13 of a total out of 18 FIT hospitals have been analyzed. For a final assessment, data covering all FIT hospitals over the entire evaluation period have to be evaluated. The scientific use of claims data from SHI funds for the evaluation of new health care concepts has been established during the last years including analysis and reporting standards (35, 36). However, validity of information on diagnoses in claims data can be a potential issue, especially regarding outpatient data (41). However, please note that due to the CG design of the study, we expect potential biases of diagnosis quality to affect each, IG and CG to equal amounts. While claims data offer essential information and have the great advantage of being less prone to selection bias (42), they do not contain preference-based and patient-centered information such as symptom severity or functional level measures. Hence, it is important to stress that this study cannot evaluate treatment success in FIT programs *per se*.

In order to close this gap and gain such information, the complementary evaluation project PsychCare has been established. This project is conducted in 10 FIT hospitals and consecutive controls and will give access to patient-reported outcomes and patient-reported experience measures by means of questionnaires and qualitative surveys. First results by another research group implementing a mixed methods design suggest that changes caused in FIT hospitals are rated mostly positive by patients, while relatives stated to fear a certain extra effort caused by increased outpatient treatment. Employees in FIT hospitals, especially in nursing professions, described to have a higher work load in response to the initiation of the FIT program (43).

## Conclusions

The indirect aim of FIT programs is to develop a system where the treatment can be adjusted flexibly to the patient in contrast to adjusting the patient to the treatment. In accordance with our hypothesis, the meta-analyses presented here suggest positive effects for models of care based on global treatment budgets for patients with mental disorders. Results point toward a shorter duration of inpatient treatment and a trend toward a reduced duration of sick leave days in FIT hospitals with a preexisting FIT–like structure in the pre-period. Moreover, the results suggest that (a) preexisting contracts are likely to have facilitated the transition into the new FIT environment and (b) implementation of FIT programs in hospitals without such a pre-contract demands for a certain transition period until positive effects will be visible. Further reports will answer the question if positive effects of intervention observed so far will persist on a long-term perspective. If FIT hospitals in psychiatric care continue to be efficient compared to routine care, this evaluation will provide arguments for a new structuring of routine care for patients with mental disorders in Germany.

## Data Availability Statement

The data supporting the findings of the study is provided by more than 70 participating SHI funds. These data are not publicly available and were used under license for the current study.

## Ethics Statement

The ethical committee of the University of Magdeburg has been notified. The ethical committee stated in April 2016 that no vote is necessary, as the EVA64-study is an analysis of anonymous data.

## Author Contributions

FB wrote the manuscript, conceptualized, and conducted the statistical analyses. OS conceptualized the statistical analyses. AN coordinated the study and defined the outcome parameters. MS contributed substantially to data handling, selection of control hospitals, and evaluation of outcome parameters. RK contributed substantially to the economic outcomes and patient–individual matching. SM contributed substantially to the data management. ES was responsible for data management. DH was responsible for economic outcomes. AP advised substantially in regard to psychiatric care and outcome parameters. JS was principal investigator and responsible for the description of the study design and effectivity estimation. All authors have read and commented on the manuscript and approved the final version of the manuscript.

### Conflict of Interest

The authors declare that the research was conducted in the absence of any commercial or financial relationships that could be construed as a potential conflict of interest.

## Abbreviations

CG: control group
DiD: difference in difference
ICD-10: International Classification of Diseases, 10^th^ revision
IG: intervention group
PIA: psychiatric outpatient department
SGB: Social Security Code (Sozialgesetzbuch)
SHI: statutory health insurance.
